# Advances in fermented foods revealed by multi-omics: A new direction toward precisely clarifying the roles of microorganisms

**DOI:** 10.3389/fmicb.2022.1044820

**Published:** 2022-12-14

**Authors:** Haisu Shi, Feiyu An, Hao Lin, Mo Li, Junrui Wu, Rina Wu

**Affiliations:** ^1^College of Food Science, Shenyang Agricultural University, Shenyang, China; ^2^Liaoning Engineering Research Center of Food Fermentation Technology, Shenyang Agricultural University, Shenyang, China; ^3^Shenyang Key Laboratory of Microbial Fermentation Technology Innovation, Shenyang Agricultural University, Shenyang, China

**Keywords:** multi-omics, fermented foods, exploration of microbial communities, health, fermentation mechanisms, flavor

## Abstract

Fermented foods generally comprise a complex micro-ecosystem with beneficial microbiota, functional products, and special flavors and qualities that are welcomed globally. Single-omics analysis allows for a comprehensive characterization of the main microbial factors influencing the function, flavor, and quality of fermented foods. However, the species, relative abundance, viability, growth patterns, and metabolic processes of microorganisms vary with changes in processing and environmental conditions during fermentation. Furthermore, the mechanisms underlying the complex interaction among microorganisms are still difficult to completely understand and analyze. Recently, multi-omics analysis and the integration of multiple types of omics data allowed researchers to more comprehensively explore microbial communities and understand the precise relationship between fermented foods and their functions, flavors, and qualities. Multi-omics approaches might help clarify the mechanisms underpinning the fermentation processes, metabolites, and functional components of these communities. This review clarified the recent advances in the roles of microorganisms in fermented foods based on multi-omics data. Current research achievements may allow for the precise control of the whole industrial processing technology of fermented foods, meeting consumers’ expectations of healthy products.

## Introduction

With the current increasing demand of consumers for healthy foods, fermented foods with beneficial microbiota, nutrients, and healthy functions have attracted increasing attention. The consumption of fermented foods has had a long history and has spread worldwide. Although many kinds of fermented foods exist, industrialized fermented products are limited; however, with the development of the fermentation theory, meta-omics technology, and the integration of multiple types of omics data, the mysterious world of microbes can be revealed.

The application of multi-omics in fermented foods may offer an opportunity to precisely discover the changes in the microbiota during fermentation, the interactions involved, the link between microorganisms and functional components, flavor substances, and deleterious inhibition, among other aspects. The key points of the processing control of fermented foods include the types of starter cultures, the microbial communities at each fermentation stage, and various operational conditions. The application of multi-omics replaces the single-cell omics approach for the analysis of microbial communities, allowing for a comprehensive understanding of the dynamic microbial changes in the food fermentation process. It is helpful to characterize the microbial community and species succession during different fermentation stages, evaluating the influence of the external environment on the microbial community, ultimately enhancing the fermentation conditions. Understanding the directional selection of a microbial community is helpful in finding ways to improve the function and added value of fermented foods. In addition, the multi-omics approach can allow for accurate analysis of the mechanism underlying the complex interactions among microorganisms, which is conducive to improving the quality, flavor, and safety of fermented foods and boosting bioactive metabolite levels essential for human health. Furthermore, the multi-omics approach has potential application for unraveling the relationship between the quality and safety of fermented food products as it involves cell data acquisition and the identification of specific genes and proteins of harmful microorganisms. Moreover, preferential starter cultures can be ascertained in the industrial process of fermented foods using the multi-omics approach. In summary, multi-omics will serve as a comprehensive processing technology, helping produce healthy fermented foods that meet consumers’ demands.

Herein, the roles of microbial communities in fermented foods were clarified *via* multi-omics strategies. Then, the relationship between fermented foods composed of complex microorganisms and their functions, flavors, and qualities was revealed using a multi-omics approach. Furthermore, the mechanism underlying the fermentation process, metabolites, and functional components was analyzed. Finally, innovation of the industrialized production mode of fermented foods was prospected based on multi-omics insights.

## The roles of microorganisms in fermented foods by multi-omics

### Disadvantages of single-omics approaches

Single-omics analysis can only describe a certain biological process and cannot analyze microbial metabolic differences or the complex mechanisms underlying their interactions during fermentation. It represents a single result at the nucleic acid, protein, or metabolite levels and cannot fully represent proteomes or metabolomes under different transcriptional conditions (Kaster and Sobol, 2020). In addition, it cannot identify low-abundance microorganisms or their roles in fermented foods, even obscuring certain interactions between microbial communities (Afshari et al., 2020a). There is a lack of effective data supporting the signal transductions, interaction networks, growth regulations, inter-species interactions, and phenotypic predictions of diverse microorganisms in naturally fermented foods. For instance, important coding genes or the enrichment of pathways discovered by metagenomics do not necessarily contribute to the changes in corresponding functional molecules. At the same time, single-omics analysis, such as metagenomics, cannot distinguish between living and dead cells, thus interfering with subsequent analyses of the effects of microbial interactions on cell metabolites (Kaster and Sobol, 2020). Moreover, metagenomics based on gene sequencing has inherent limitations, including the inability to directly determine the functional activity of microorganisms and the difficulty in identifying molecules performing critical functions (Franzosa et al., 2014). Finally, single-omics analysis cannot bypass the issue of whether whole microbial communities are equivalent to the microorganisms that produce active components during fermentation. For example, transcriptomics analyzes gene expression levels, whereas proteomics aims to explore the functions and key enzymes of microorganisms at the protein level, neither of which can fully yield integrated data on the relationship between microbial communities and the effects thereof on active components (Yang et al., 2020).

### Advantages of multi-omics application

**FIGURE 1:**
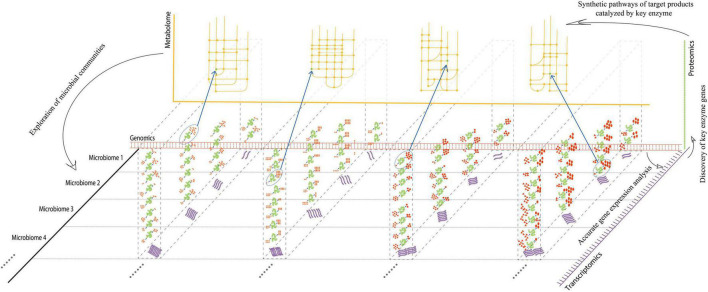
Advantages of multi-omics analysis at multi-dimensions. Red double-chain structures indicated “genomics,” while purple single-chain structures indicated “transcriptomics,” originating from different microorganisms split by a horizontal dotted line. In each microbiome, the transcription level of the corresponding gene may be different. Proteins or enzymes (green) translated by RNA at different transcriptional levels may have different activities, catalyzing different substrates for products (red). The bottom left corner of each enzyme indicated the substrate, and the top right-hand corner indicated the product. The numbers of substrates and products indicated different catalytic activities for each enzyme. The enzymes with the highest activities, which participated in the corresponding pathways, were circled by dotted ellipses. Synthetic pathways (yellow) of target products can be analyzed by key enzymes discovered at the metabolic level. Exploration of microbial communities can be achieved by each omic from every dimension.

Multi-omics can span the multi-layer analysis of gene and protein expression, as well as metabolite differences of all microorganisms in the environment, consequently yielding a large amount of data (Figure 1). It is well-known that in systems biology, multi-omics jointly “open up” multiple levels of analysis, allowing an exploration of the development, differentiation, signal transduction, and interaction network between cells in response to environmental changes. Furthermore, multi-omics can provide effective regulatory targets for stress disturbance in response to environmental changes, including the regulation of the internal metabolic pathways of the host (Kopczynski et al., 2017). Specifically, multi-omics is applied to fermented foods research: culturology separates living bacteria from traditional fermented foods; genomics, transcriptomics, and proteomics analyze the structure, regulation, and expression of microbial functional genes in traditional fermented foods; macrogenomics and metabolomics clarify the diversity of microbial functional genes and their metabolites. Multi-omics analysis can integrate multi-level analyses and has a multiplier effect on the screening, evolutionary succession, functional gene screening, expression, and metabolic network mining of microbes in fermented foods.

Multi-omics is regarded as the mainstream way of analyzing fermented food microorganisms and has marked advantages of “1 + 1 > 2” in analyzing microbial succession, determining functional genes, and exploring the metabolic network of active components (Figure 1). However, research on microbial succession and metabolic mechanisms of fermented foods using multi-omics is still in its infancy. On the one hand, differences in the production process of traditional fermented foods, spatial heterogeneity, dynamic variability in the fermentation environment, and uneven distribution in the microbial communities lead to the evolution of microorganisms at different growth points due to the interaction of different mechanisms, resulting in a variety of phenotypes, implying the lack of representativeness of multi-omics approaches. On the other hand, the speed of the research and development on sequencing and mass spectrometry exceeds the development of analysis software or databases, resulting in a large amount of data that cannot yet be fully analyzed.

### Multi-omics-based exploration of microbial communities in fermented foods

The production of fermented foods needs the coordinated action of a plethora of microbial communities. The analysis of species, metabolic pathways, and interactive relationships of these microbial communities is conducive to the directional control of microbial species in the fermentation process, therefore improving production efficiency and the flavor of fermented foods, as well as ensuring the safety thereof.

The coordination of the microbial communities of fermented foods can be achieved by multi-omics from virtually every dimension (Figure 1). It can provide an overall view of which microorganisms are present in a community, how they behave, interact, and what the phenotypic manifestations of this complex arena are, which is conducive to studying traditional microbiology in the fermented food industry. From 2011 to 2021 (Supplementary Figure 1), the number of studies published on the application of multi-omics in fermented foods increased, indicating a growing interest in multi-omics-based research on fermented foods. This is because multi-omics can help researchers systematically analyze changes in the factors that regulate the whole fermentation process. This includes the exploration of all microorganisms involved in fermentation to master and control the regularity of dynamic changes in the microbiota at each point of fermentation. Another goal is to unearth the mechanisms underlying the effects of microbial communities on the quality, flavor, and safety of fermented foods under various fermentation conditions.

Multi-omics approaches have far-reaching implications for fully exploring food microbial resources and improving the quality of fermented foods. In the past, owing to technological limitations, researchers thought that fermented foods were products of fermentation by a single or several strains. However, with the recent development of multi-omics, numerous studies have focused on food microbial communities. Accordingly, a large number of new and previously unknown microbial communities have been discovered in fermented foods (Supplementary Table 1). The source classification and geographical distribution of microorganisms in food fermentation in different countries are shown in Supplementary Figure 2. A total of 33 countries were found as source countries. There were 17 countries with more than 15 species, accounting for 52% of the total identified species. India, Korea, Nepal, China, Indonesia, Pakistan, Japan, and Bangladesh were the top eight source countries.

The exploration of microbial communities has played a fundamental role in food fermentation, in which complex microbial communities are inherently involved in the quality, as well as safety, of the product (Bokulich et al., 2016). Using metaproteomics, Xie et al. (2019a,b) found 1,415 microbial protein clusters in soybean paste; their Illumina MiSeq results showed a high diversity of microbial communities in soybean paste. Alcohol dehydrogenase produced by *Tetragenococcus* sp., and family Lactobacillaceae, genus *Leuconostoc* sp., was highly abundant in naturally fermented soybean paste. The strains of the species thereof played a key role in forming alcohol flavor components, as evidenced by a combination of metaproteomics and metabonomics (Xie et al., 2019a,b). Lee et al. (2020) revealed that metagenomic, metatranscriptomic, and metabolomic analyses provided information on the preferred carbon sources of individual microorganisms, including the various genes and intermediate metabolites involved in the kimchi fermentation using these carbon sources. Afshari et al. (2020b) reported that metagenomic and metabolome analyses revealed qualitative and semi-quantitative differences in microbiota metabolites between different types of cheese. They also found that the presence of two compounds (3-hydroxypropanoic acid and *O*-methoxycatechol-*O*-sulfate) in artisanal cheese had not previously been reported in any type of cheese. Their integrative analysis of multi-omics datasets revealed that highly similar cheeses, identical in age and appearance, could be distinctively clustered according to the cheese type and brand (Afshari et al., 2020b). The above results reveal that multi-omics can enhance the understanding of which microbes are present in the fermentation process, as well as improve the understanding of the complex interactions among microbes and overall microbial activities.

### Multi-omics insights into microbial succession at different fermentation stages

The microbial succession of fermented food plays a key role in its quality and safety. In the early stage of food fermentation, bacteria are in the growth phase, and the number of microbial species is relatively small. However, the number of microbial species greatly increases during the middle and late fermentation stages, when harmful bacteria, such as *Staphylococcus aureus*, *Listeria monocytogenes*, and *Staphylococcus equorum*, also begin to proliferate (Supplementary Table 2). However, there is still a lack of systematic research on the regulation of microbial succession and its mechanism in fermented food.

Multi-omics data can reveal microbial succession in different fermentation stages. Dugat-Bony et al. (2015) reported that metagenomics and macro transcriptomics could be combined to reveal the dominant microbial species in the cheese fermentation process, including the effect of their interactions on milk-based product degradation. On day one of their study, *Lactococcus delbrueckii* subsp., lactis and *Kluyveromyces lactis* were the most active species, both of which consumed lactose quickly during their early stage of maturity; the produced lactic acid was rapidly consumed by *Debaryomyces hansenii* [lactose consumption increased from day 1 (1%) to day 14 (9%)] and *Geotrichum candidum* (the dominant strain on day 7) (Dugat-Bony et al., 2015). *Corynebacterium casei* and *Hafnia alvei* were detected on day 31. In the first 2 weeks of maturation, the dominant species, *L. lactis* and *K. lactis*, were gradually replaced by *C. casei* (Dugat-Bony et al., 2015). According to Bertuzzi et al. (2018) metagenomics and metabolomics could be combined to confirm that *D. hansenii* and *G. candidum* were the dominant strains during the first mature stage of surface-ripened cheese. However, they were replaced by *Brevibacterium linens* and *Glutamicibacter arilaitensis* during a later stage (Bertuzzi et al., 2018). Ruggirello et al. (2018) using Illumina HiSeq sequencing and gas chromatography-mass spectrometry (GC-MS), found that *L delbrueckii* subsp., lactis was the most abundant microorganism during cheese production and early ripening but began to decline significantly after 30 days of ripening and was later no longer detected. He et al. (2020) found that the microbial communities of Chinese sauerkraut were dominated by the *Serratia* and *Pseudomonas* genera during the early stages of fermentation and by the family Lactobacillaceae, genus *Lactobacillus* during the later stages. He et al. (2020) also detected a total of 86 volatile compounds in sauerkraut samples using genomics and metabonomics. Of these compounds, 13 were significantly positively correlated with lactic acid bacteria, whereas 11 were significantly negatively correlated with *Pseudomonas* sp. (He et al., 2020). These studies analyzed microbial succession using multi-omics, which can further reveal the flavor profiles, functional components, and their regulation in fermented food at each fermentation stage.

## Multi-omics insights into the relationship between fermented foods composed of complex microorganisms and their function, flavor, and quality

### Function

Some unique components contained in functional fermented foods are generally considered bioactive compounds produced by microorganisms, which positively improve human health and physical function. At present, single-omics analysis is mainly used to reveal the relationship between the functionality of fermented foods and their microbial communities; however, most results are unsatisfactory. Applying the principles of multi-omics can improve the identification of bioactive compounds in fermented foods, as well as their safety and reliability. Sugahara et al. (2017) analyzed the differences between live and heat-inactivated *Bifidobacterium breve* in regulating host immunity, intestinal metabolism, and intestinal gene expression using transcriptomics and metabolomics. They believed that both cells had the potential to regulate immunity, inhibit the production of pro-inflammatory cytokines in splenocytes, and affect intestinal metabolism. Nonetheless, these functions were presented more significantly in living cells than the inactivated ones (Sugahara et al., 2017). The combination of omics enables the detection of specific genes that produce specific active constituents in fermented foods, in turn improving the functions of fermented foods (Cocolin et al., 2018). Previous studies have shown that complex bio-zone systems cannot be accurately identified by single-omics analyses, such as genomics, which explains the changes in gene abundance and interactions between microbial colonies, but not the potential links to phenotypes (Amer and Baidoo, 2021; Ferrocino et al., 2022). Therefore, a multi-omics approach is adopted to systematically track specific genes that produce specific active constituents in fermented foods (Cocolin et al., 2018); these data are then combined with the final phenotype using transcriptomes and proteomes, further enhancing the understanding of fermented food systems. Wang et al. (2023) established a murine model of hyperuricemia to explore the effective treatment ability of *Bacillus subtilis*-fermented *Astragalus* using a multi-omics approach, which revealed that the abundance of butyrate-producing bacteria (*Odoribacter splanchnicus* and *Collinsella tanakaei*) and probiotics (*Lactobacillus enterocolitica* and *Bacillus mycoides*) increased significantly during fermentation, thereby effectively reducing kidney inflammation and regulating the expression of uric acid transporters to treat hyperuricemia. Using a multi-omics approach, it is more affordable to identify genes and gene products responsible for the functional metabolites in fermented foods, yielding highly active metabolites for human health (Lee et al., 2021).

### Flavor

The formation mechanism of characteristic flavor components in fermented food can be revealed by multi-omics, and functional microorganisms are screened to guide the production process of fermented foods. *Via* the analysis of the differences in microecological diversity, genes, and metabolic levels by multi-omics, the transcription and expression of microbial genes related to the formation of characteristic flavor components can be clarified, and key biomarkers for the metabolic regulation of fermentation flavor components are characterized. Wu et al. (2022) analyzed the regulatory relationship between differential genes and secondary metabolite biosynthesis in fermented soybean by transcriptomics, proteomics, and metabolomics. They found a total of 130 upregulated metabolites and 160 downregulated proteins related to fermented soybean flavor (Wu et al., 2022). Multi-omics can greatly promote the understanding of microbial evolution, physiology, and metabolic pathways in fermented foods, as well as predict the formation of expected and undesired flavors according to conditions of flavor formation, which are affected by each strain. Multi-omics can also explain the influence of microorganisms on the flavor of fermented foods *via* the catalysis of enzyme–substrates and their interaction at the species level. Karaduman et al. (2017) and Lu et al. (2018) used proteomics and metabolomics as powerful methods for real-time *in situ* detection and quantitative analysis of colony metabolites. By doing so, they could detect and guide the real-time changes of microorganisms and metabolites in fermented dairy products (Karaduman et al., 2017; Lu et al., 2018). Hu et al. (2021) clarified the relationship between fungal communities and non-volatile flavor compounds during solid-state batch fermentation of green tea using genomics and metabolomics. The dominant fungal strain in green tea fermentation was *Aspergillus*, which can produce abundant hydrolases that hydrolyze cellulose, pectin, and protein in the tea cell wall, forming soluble carbohydrates, amino acids, soluble pectins, and other compounds conducive to the taste and organoleptic properties of green tea (Hu et al., 2021). Applying a combination of genomics and metabolomics, Unno et al. (2021) found that lactic acid was positively distributed and accounted for 41% of the total variance on surface-ripened mold cheeses and smear cheese. Notably, 47.7% of ketones and alcohols were produced by specific bacteria (*Pseudoalteromonas* sp. and *Marinomonas* sp.) (Unno et al., 2021).

### Quality

There are individual metabolic differences and complex interactions in microbial communities in fermented food, which cannot ensure the stable quality and food safety of final fermented products (Montel et al., 2014). Multi-omics can systematically and deeply analyze the beneficial, pathogenic, and spoilage-related strains during fermentation. Fermented foods are less likely to be spoiled under fermentation conditions, mainly due to the correct control of the reproduction of pathogens by factors such as fresh food materials, fermentation time, and pH. Song et al. (2020) divided kimchi into two groups: one cultured using a starter culture of kimchi, sauerkraut, and garlic was successfully fermented; the other cultured using a starter culture of ginger and red pepper could not support fermentation, revealing that different starter sources significantly differ in terms of dominant microbes and their metabolites. Thus, the microbial communities determined the final quality of fermented food (Song et al., 2020). Medina et al. (2016) indicated that the quality of natural green olive was better during the early and middle stages of fermentation, but spoilage microorganisms such as *Pseudomonas*, *Propionibacterium*, *Modestobacter*, *Rhodovibrio*, and *Salinibacter* appeared during a later fermentation stage. Wu et al. (2019) combined proteomics and transcriptomics, revealing that the decrease of milk pH (pH 5.5) during fermentation inhibited the expression of glutamate differential proteins by 0.43-fold. In contrast, it upregulated the expression of two key proteins (locus: T303-05420 and T303-05425) involved in cysteine catabolism by 4.25- and 7.26-fold, respectively, thus affecting the milk quality (Wu et al., 2019).

## Multi-omics insights into the mechanism underlying the fermentation process, metabolites, and functional components

Traditionally, in fermented foods, the main contribution of bacteria is to produce flavor compounds; yeast produces alcohol and low levels of flavor compounds, and mold decomposes macromolecules. Although this understanding is reasonable, it should also be noted that this understanding is based on the physiological and metabolic characteristics of some single-cultured microorganisms. There is a serious lack of understanding of the functions of non-isolated cultured microorganisms. More importantly, there is a lack of a systematic analysis of the mechanism underlying microbial interaction in complex fermentation systems and its effect on fermentation processes, metabolites, and functional components.

Multi-omics can be used as the main way of analyzing the correlation between microbial communities, gene metabolites, and the interaction mechanism in fermented foods, and characterize the regulation mechanism underlying microbial communities of fermented foods during fermentation as well as the metabolites and functional components thereof at multiple levels (Sattin et al., 2016). Multi-omics is bound to become the main research field of fermented foods to build a predictive model of microbial communities by monitoring the dynamic changes of microorganisms, their differential gene expressions, and their metabolite compositions in real-time. Zhang et al. (2020) revealed the functional microorganisms during the first stage of Pixian soybean paste fermentation by amplicon sequencing and proteomics. They found three strains secreting peptidase and producing amino acids, which can induce polypeptide degradation in hypertonic fermentation (Zhang et al., 2020). Hu et al. (2021) used genomics and metabolomics to explore the effect of fungal succession on the content of various non-volatile flavor compounds in fermented dry green tea. The alkaloid level decreased by 37.50%, and the catechin level decreased from 7969.98 ± 346.36 to 233.98 ± 20.29 μg/g (Hu et al., 2021). Settachaimongkon et al. (2016) used headspace solid phase microextraction (SPME)-GC/MS and metabonomics to reveal the significant effect of sublethal precultured *Lactiplantibacillus plantarum* WCFS1 on the metabolite spectrum of yogurt. The addition of precultured *L. plantarum* impaired the survival of *Lactobacillus delbrueckii* (Settachaimongkon et al., 2016). Piddocke et al. (2011) clarified the effect that the addition of multicomponent protease had on the metabolism of brewer’s yeast *via* transcriptomic and metabolomic analysis. In addition, environmental factors are considered in the multi-omics analysis to predict the physiological characteristics and microbial succession in a specific environment so that it can more objectively reveal the interactions of microbial communities in fermented foods. Stellato et al. (2015) believed that there were differences in metabolic pathways between environmental samples from surfaces and tools, and the different types of cheese samples produced by the same factory. The persistence of microorganisms in the environment may resist the development of potentially harmful species that may contaminate cheese and adversely impact product quality (Stellato et al., 2015).

Multi-omics has gradually gained popularity in the field of the fermentation mechanisms of various fermented foods. However, many hindrances appear at present. The primary problem for the mechanisms underlying the fermentation process, metabolites, and functional components to characterize microbial communities of fermented foods includes how to analyze and explore a large amount of multi-omics data and how to effectively use the results thereof to objectively form the fermentation system. Secondly, multi-omics are comprehensively used to analyze the assembly mechanism of microbial communities in fermented foods and reveal the influencing factors of community assembly and its effect on later fermentation. In addition, the establishment of supporting databases and multivariate statistical analysis models also need to be timely followed up on for a large amount of obtained multi-omics data. Finally, traditional isolation and culture of microbes is the most intuitive way to verify the prediction of microecological communities by multi-omics data, which is necessary for the annotation of new genes, as well as the functional characterization and physiological identification of species. The results of multi-omics combined with traditional isolation and culture are currently still missing a link in systems biology (Jansson and Baker, 2016).

## Innovation of industrialized production mode of fermented food *via* multi-omics

The Multi-omics Database of Microbes in Fermented Foods (MDMFF) should be further developed for application in the fermented foods industry (Lee et al., 2020). Microbial resources are the core to updating the industrial production mode of future fermented foods. The establishment of fermented food culture collection can protect and preserve beneficial strains, effectively avoiding their extinction and inheriting the valuable microbial resources known for thousands of years in the traditional fermentation of foods. Furthermore, MDMFF can offer quality genomes, metataxonomes, metagenomes, metatranscriptome sequences, metaproteomes, and associated metabolome information of fermented food-associated bacteria, archaea, and eukaryotic microorganisms. Furthermore, the database will include several analytical tools for multi-omics analyses by an input query in the database. Lee et al. (2020) recently developed an Omics Database of Fermentative Microbes, integrating comprehensive omics information from fermentative microorganisms at the World Institute of Kimchi. It provides basic information to evaluate microbial strains isolated from fermented foods as candidate starter cultures in terms of the fermentation processes, qualities, flavors, and sensory properties. Moreover, multi-omics can couple the interactions between microorganisms in fermented foods with their quality and safety, helping promote healthy production management at the industrial level (Ferrocino et al., 2022). Siren et al. (2019) used genomics, proteomics, and metabolomics to explore the interactions between microbial communities and the factory environment during wine fermentation by *Saccharomyces cerevisiae* and *Oenococcus oeni*. Suitable probiotics were also used to promote the formation of specific aromas, which was conducive to the production of industrial wine (Siren et al., 2019).

Fermented food production should be standardized to predict and control the fermentation process using these multi-omics data. Multi-omics can be used to accurately select the cultures required for the formation of qualities, flavors, and sensor properties of fermented foods. By connecting genomic characteristics with phenotypic output and exploring the metabolic diversity of starter cultures, the impact of a single strain on the qualities, flavors, sensor properties, and metabolic pathways of fermented food can be understood to customize the starter mixture to meet the needs of specific fermented foods in the industry. Multi-omics can also be used to optimize the control of fermented foods under different processing and storage conditions to accurately determine the detailed parameters of various processes in each fermentation stage and ensure the optimal quality, flavor, aroma, nutrition, and safety of fermented foods. Janssen et al. (2020) and Franciosa et al. (2021) conducted multi-omics analyses of fermented sausages, revealing the role of microbial communities in the production chain during fermentation. These studies identified specific metabolic pathways during sausage fermentation and provided a basis for the growth of local microbiomes, thereby improving and controlling the industrial fermentation process and enhancing product quality. Alessandria et al. (2016) used genomics and proteomics to explore the microbial communities of Italian hard cheese, analyze the mechanism underlying the antagonism and coexistence between starter lactic acid bacteria and microbial colonies, and explain the taste, aroma, and texture of that cheese, so as to promote safe production in the cheese industry. These processes are particularly helpful for developing high-quality starter cultures and new products with higher qualities, improved sensor properties, unique flavors, and specific functions for the future fermented food market.

## Conclusion

The roles of microorganisms profoundly affect the quality, flavor, and safety of fermented foods. More microbial communities can be investigated using multi-omics, rather than single-omics, to reveal new findings between fermented foods composed of complex microorganisms and their functions, flavors, and qualities. Analyzing the mechanism underlying the fermentation process, metabolites, and functional components using multi-omics provides in-depth technical support for the industrialized production mode of fermented food.

## Author contributions

HS: conceptualization and roles/writing – original draft. FA: investigation, data curation, and resources. HL: software and formal analysis. ML: validation. JW: project administration. RW: supervision, writing – review and editing, and funding acquisition. All authors contributed to the article and approved the submitted version.
