# Supplementary material for: Advances in fermented foods revealed by multi-omics: A new direction toward precisely clarifying the roles of microorganisms
Source: Front Microbiol. 2022 Dec 14;13:1044820. doi: 10.3389/fmicb.2022.1044820 (PMC9794733; doi:10.3389/fmicb.2022.1044820)
Supplement: Supplementary file 1 [file Data_Sheet_1.docx]

Supplementary Material

# Supplementary Figures and Tables

## Supplementary Figures

**
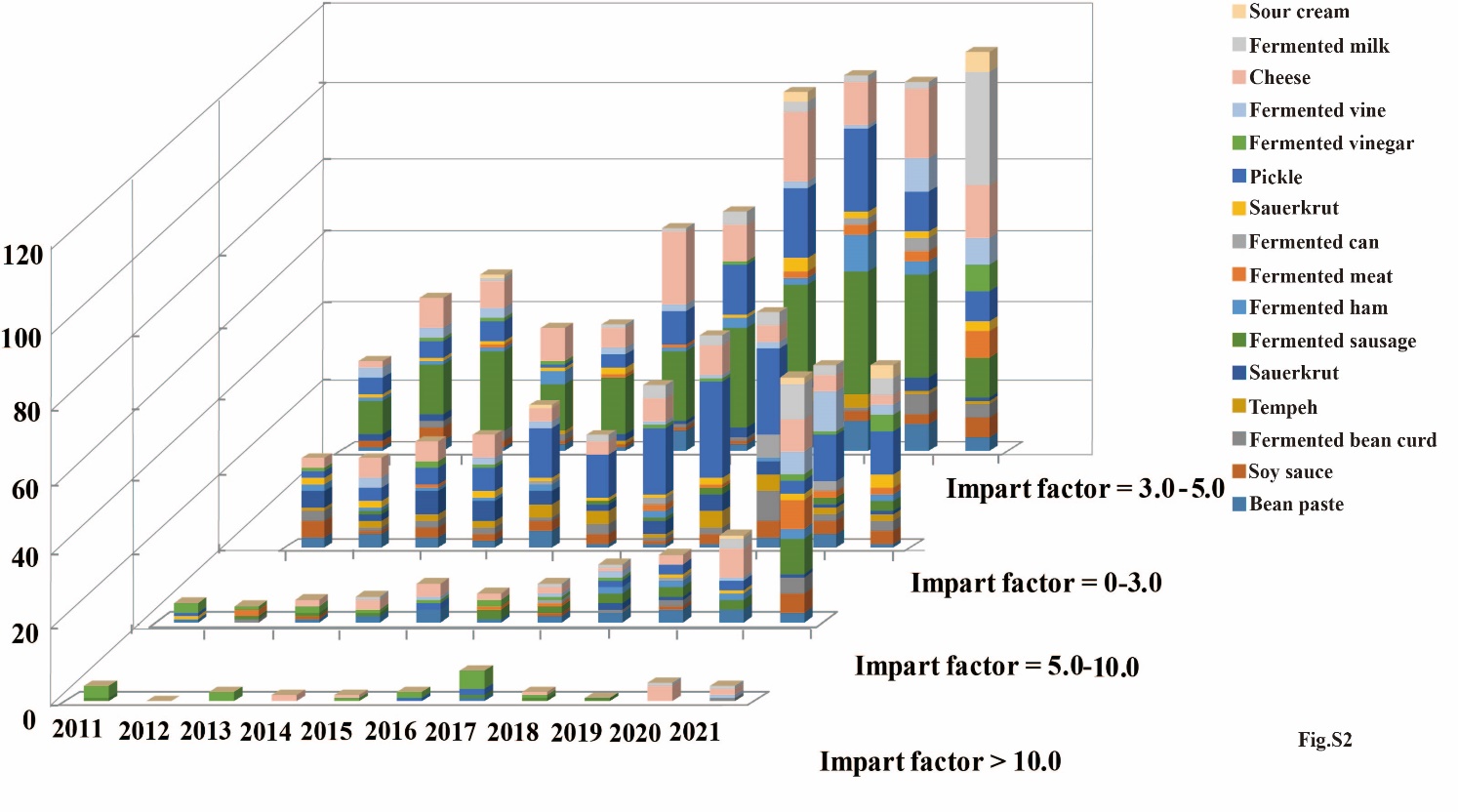
**

**Supplementary Figure 1.** The number of published articles in the field of multi-omics on fermented foods in recent decades.

**
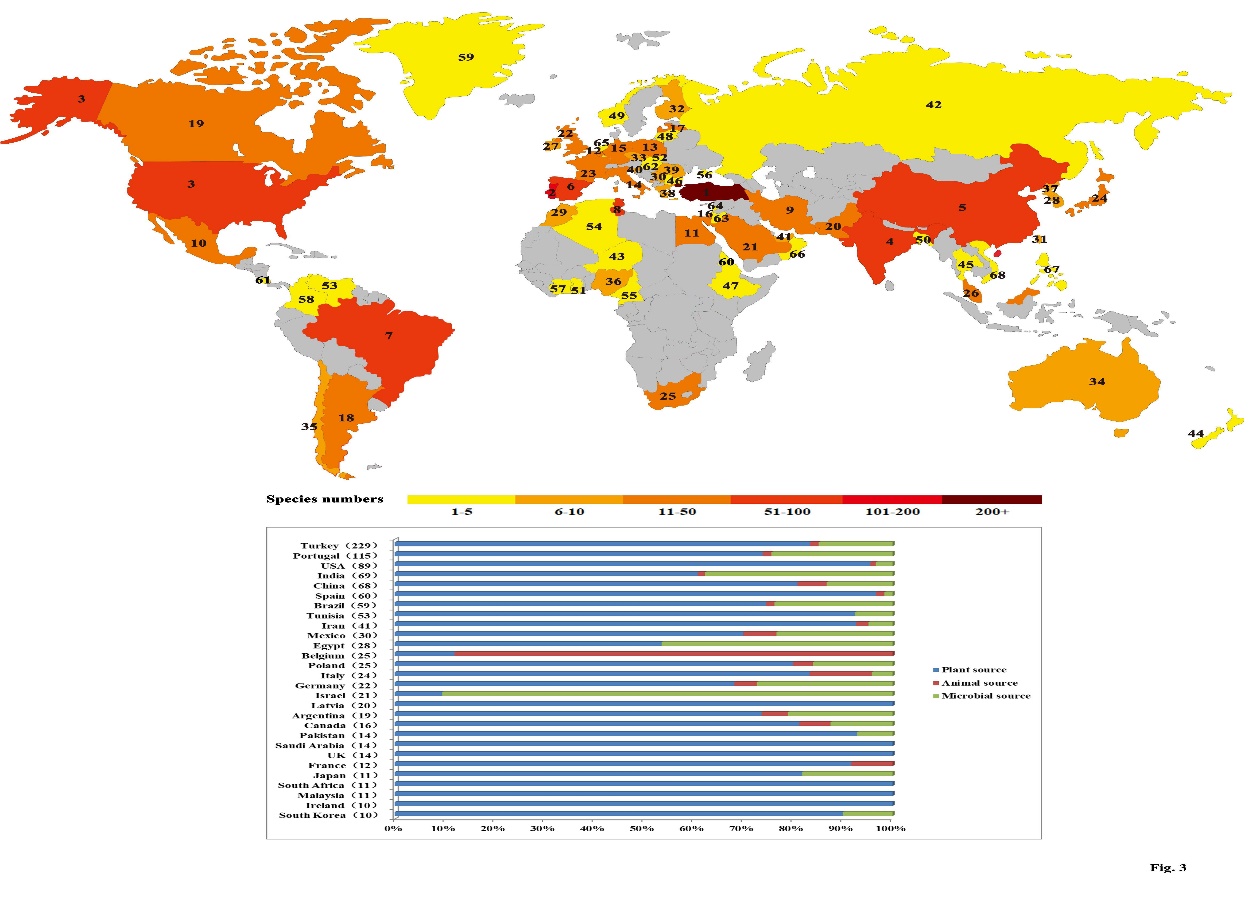
**

**Supplementary Figure 2.** Overview of geographical distribution of microorganisms in food fermentation by multi-omics analysis. Number of species in brackets; only those countries that had 10 species or greater across all countries are indicated. 1: India, 2: Korea, 3: Nepal, 4: China, 5: Indonesia, 6: Pakistan, 7: Japan, 8: Bangladesh, 9: Bhutan, 10: Vietnam, 11: Malaysia, 12: Ghana, 13: Sri Lanka, 14: Togo, 15: Benin, 16: Spain, 17: Australia, 18: Mongolia, 19: Portugal, 20: Thailand, 21: Philippines, 22: Brazil, 23: Kenya, 24: Russia, 25: America, 26: Sudan, 27: Tanzania, 28: Canada, 29: Congo, 30: Singapore, 31: Finland, 32: The Netherlands, 33: Turkey.

## Supplementary Tables

**Supplementary Tables 1** Traditional recognition and multi-omics based recognition of microflora in fermented foods^*^

| **Fermented foods** | **Subsidiary fermented foods** | **Traditional recognition of microflora** | | **Multi -omics based recognition of microflora** | | **References** |
| --- | --- | --- | --- | --- | --- | --- |
|  |  | **Quantities** | **Microbial communities** | **Quantities** | **Microbial communities** |  |
| **Fermented dairy products** | Yogurt and sour cream | 4 | *Streptococcus salivarius, thermophiles, Lactobacillus delbrueckii, Lactobacillus delbrueckii* subsp*. bulgaricus* | 41 | *Lactobacillus acidophilus, Streptococcus salivarius, Streptococcus thermophilus, Penicillium, Rhizopus, Fusarium, Mucor, E.casseliflavus, E.italicus, E.faecium, Limosilactobacillus fermentum, Lacticaseibacillus paracasei, Lactiplantibacillus plantarum, Lactiplantibacillus curvatus, Lactococcus delbrueckii subsp. lactis, Lactococcus garvieae, Leuconostoc mesenteroides, Leuconostoc garlicum, Lactobacillaceae lactis, Lactococcus raffinolactis, Acinetobacter lwoffii, Lactococcus chungangensis, Acetobacter cibinongensis, Acinetobacter johnsonii, Paralactobacillus.acidilactici, S.thermophiles, Weissella hellenica, brevicompactum, Aspergillus, Penicillium, Curvibacter, Acinetobacter, Chryseobacterium, Pseudomonas, Carnobacterium, Streptoverticillium mobaraense, Streptococcus thermophilus, Lactobacillus delbrueckii* subsp*. bulgaricus, Lactobacillus delbrueckiis.*sp*, Bifidobacterium lactis* | (Han et al., 2014; da Costa et al., 2016; Yu et al., 2018; Akiyama et al., 2019; Silva et al., 2019; Ziarno and Zareba, 2020) |
|  | Cheese | 3 | *Yeast, Lacticaseibacillus casei, Lacticaseibacillus* subsp*. cremoris,* | 55 | *Lacticaseibacillus casei, Lacticaseibacillus paracasei, Listeria, Achromobacter spp, Acidovorax spp, Acinetobacter spp, Aerococcus spp, Arthrobacter spp., Brachybacterium spp, Brevundimonas spp., Carnobacterium maltaromaticum, Carnobacterium spp, Chryseobacterium spp, Curvibacter spp, Debaryomyces hansenii, Dermacoccus spp, Devosia spp, Dyadobacter spp, Enterobacter spp, Enterococcus casseliflavus, Enterococcus faecalis, Flavobacterium spp, Rhodococcus erythropolis, Geobacillus stearothermophilus, Haloanella spp, Janibacter spp, Kocuria spp, ethylobacterium spp, icrobacterium gubbeenense, Ochrobactrum spp, Pseudomonas putida, Plantibacter spp, Lacticaseibacillus rhamnosus, Paenibacillus spp, Lactiplantibacillus plantarum, Latilactobacillus curvatus, enterococci, staphylococci, Rhodococcus erythropolis, Rothia spp, Sanguibacter spp, Shewanella putrefaciens, Sphingobacterium spp, Staphylococcus equorum, anaerobiosis, Propionibacterium freudenreichii, Propionibacterium jensenii, Propionibacterium thoenii, Propionibacterium acidipropionici, Propionibacterium cyclohexanicum, Propionibacterium microaerophilum, Propionibacterium thoenii, Propionibacterium acidipropionici, Propionibacterium freudenreichii* | (Schirmer et al., 2013; Yee et al., 2014; Anna et al., 2016; Yang et al., 2020) |
| **Fermented vegetables** | Chinese fermented vegetables | 1 | *Lacticaseibacillus* | 12 phyla, 21 classes, 52 orders, 97 families, 223 genera and 348 species | *Bacillus* and unclassified *Weissella* spp. were as the predominant firmicutes bacteria, while *Chromohalobacter japonicus, Halomonas jeotgali, Pediococcus* and unclassified *Vibrio* spp. were the most abundant proteus bacteria; and *Aspergillus oryzae* was the predominant fungi. | (Liu and Tong, 2017) |
|  | Kimchi | 3 | family *Lactobacillaceae, genus Lactobacillus, Leuconostoc* and *Weissella* | 12 | Six lactic acid bacteria (LAB), *Latilactobacillus (Lc.) mesenteroides, Latilactobacillus (Lb.) sakei, Lactobacillaceae. koreensis, Lc. gelidum, Lc. carnosum, and Lc. gasicomitatum* | (Jung et al., 2013) |
| **Fermented bean products** | Soybean paste | 8 | *Cladosporium, Aspergillus, Eurotium, Mucor, Lichtheimia, Penicillium, Scopulariopsis* and *Rhizopus* | 234 genera of bacteria in 14 phyla, 76 genera of fungi in 5 phyla | Among the bacteria, sclerecta (74.77%), proteus (22.61%) and actinomycetes (2.55%) were the dominant bacteria, and *Staphylococcus* constituted the majority of sclerecta. Among fungi, ascomycetes, basidiomycetes and Zygomycetes accounted for 94.88%, 3.29% and 1.77% respectively, while mycospheres and echinomycetes accounted for the rest 0.06%. Most species came from *Ascomycetes*, unclassified bryophytes and *Debaryomyces.* | (Sun et al., 2018) |
|  | Soy sauce | 5 | *Aspergillus oryzae, Aspergillus sojae, Zygosaccharomyces rouxii, Tetragenococcus halophilus* and *Candida* | 184 bacteria genera and 116 fungi genera in genus | family *Lactobacillaceae, genus Lactobacillus Weissella and Pediococcus, Staphylococcus, Bacillus, Torulaspora delbrueckii, Pichia guilliermondii, Aspergillus oryzae and Aspergillus sojae* | (Sulaiman et al., 2014; Song et al., 2015; Liang et al., 2019) |
|  | Tungrymbai and bekang (naturally fermented soybean foods of India) | NA | NA | 428 isolates of *Bacillus* | *B. licheniformis, B. pumilus, B. subtilis, Bacillus brevis, Bacillus coagulans, B. circulans, B. licheniformis, B. pumilus, B. sphaericus, Lysinibacillus fusiformis* | (Chettri and Tamang, 2015) |
|  | Fermented mung beans | NA | NA | 1264 genus and 586 species | family *Lactobacillaceae, genus Lactobacillus and Weissella, Porphyromonadaceae, Butyricimonas virosa, Exiguobacterium mexicanum, B. virosa, Bifidobacterium, Carnobacterium Collins,*  *Desemzia, Facklamia, Globicatella, Ignavigranum.* | (Chao et al., 2013) |
| **Fermented meat products** | Italian Cinta Senese sausages | 2 | *Staphylococcus xylosus* and *Lactobacillaceae sakei* | At least 32 genera | The bacterial communities were mainly composed of sclerebia (96.2%), especially *Staphylococcus* and family *Lactobacillaceae, genus Lactobacillus*, *Leuconostocaceae, Lactobacillaceae* accounting for 91.6% of the total prokaryotes (63% and 28.6%, respectively), including *Xanthomonadaceae, Vibrionaceae, Pseudomonadaceae, Moraxellaceae, Halomonadaceae, Enterobacteriaceae, Streptococcaceae, Enterococcaceae, Carmobacteriaceae, Staphylococcaceae, Planococcaceae, Listeriaceae, Bacillaceae, Micrococcaceae, Corynebacteriaceae, Haloferacaceae*, et al. | (Pini et al., 2020) |
|  | vacuum-packaged smoked bacon | 2 | *Staphylococcus* and family *Lactobacillaceae, genus Lactobacillus* | 336 | *Eubacterium hallii groug, Moellerella, Pseudoalteromonas, Bifidobacterium, Escherichia-Shigella, Microbacterium, Photobacterium, Staphylococcus, Blauta, Dysgonomonas, Lactococcus, Enterococcus, Enhydrobacter, Vagococcus, Citrobacter, Streptococcus, Psychrobacter, Acinetobacter, Dermacoccus, unidentified_Mitochondria, Fusobacterium, Rahnella , Carnobacterium, Macrococcus, Kocuria, Serratia, Brochothrix, Vibrio,* family *Lactobacillaceae, genus Lactobacillus* and *Leuconostoc*, et al. | (Li et al., 2019) |
|  | Iberian dry-cured ham | 3 | *Micrococcaceae*, moulds and yeasts | 227 bacteria and 24 yeasts | *Staphylococcus equorum, Staphylococcus epidermidis, Staphylococcus hominis, Staphylococcus　 pasteuri/warneri, Staphylococcus saprophyticus, Enterococcus faecalis, Enterococcus faecium/hirae, Carnobacterum divergens, Kocuria palustris, Kocuria rhizophila, Tetragenococcus halophilus, Tetragenococcus koreensis, Tetragenococcus solitarus, Brachybacterum conglomeratum, Streptomyces flavofungini/lomodensis, Debaryomyces hansenii, Moniliella nigrescens, Cryptococcus magus, Rhodotorula mucilaginosa, Ustilago cynodontis/sparsa*, et al. | (Martinez-Onandi et al., 2019) |
|  | Salamis | 4 | *Latilactobacillus sakei, Latilactobacillus curvatus, Lactiplantibacillus plantarum, Staphylococcus xylosus* | 35 *Lactobacillus* and 21 *Staphylococcus* | *Lb.sakei, C.divergens, Lb.curvatus, Ln.carnosum, L. delbrueckii subsp. lactis, Ln.mesenteroides, St.equorum, St.xylosus, St.edaphicus, St.edaphicus, St.saprophyticus, St.vitulinus, Ln. esenteroides*, et al. | (Settanni et al., 2020) |
|  | raw meat and processed meats from equine origin | 2 | LAB and coagulasenegative *Staphylococci* (CNS) | 1567 | *Carnobacterium divergens, Latilactobacillus curvatus, Latilactobacillus sakei, Lactococcus piscium, Leuconostoc elidum* subsp*. elidum, Leuconostoc mesenteroides, Staph lococcus carnosum, Staph lococcus equorum, Staph lococcus saproph ticus, Staph lococcus similans and taphylococcus xylosus*, et al. | (Geeraerts et al., 2019) |
|  | Chinese dry-cured sausage/Chinese smoked-cured sausage | 5 | *Staphylococcus* spp. family *Lactobacillaceae, genus Lactobacillus* spp., *Weissella* spp., *Pediococcus* spp., and *Lactococcus* spp., mold, yeast | More than 47 species | *Staphylococcus, Cornebacterium, Weissella, Flavobactertum, Dcinetobacter, Drthrobacter, Pediococcus, Delftia, Lactococcus, Exiguobacterium, Lactobacillus, Myoides, Pseudomonas, Rothia, deromonas, Xanthomonas, Brochothrit, Kocuria, Psychrobacter, Chryseomicrobium, Snreptococcus, Cobetia, Pantoea, bydrogenophaga, Macrococcus, Burkholderia, Enterococcus,Vibrio, Camobacterium, Brevwundimonas, unclassified Janthinobacterrum, Photobacterium, Anonybacillus, Chryseobacterium, Stenotrophomonas, Leluconostoc, Rhizobium, Shewanella, Geobacilus, Enhydrobacter drrobacter, Tagococcus, Defluvimonas, Kurthia, Bacillus, Cirobacter, Salinivibrio iridibacillus*, et al. | (Wang et al., 2018) |
|  | Spanish-type chorizo | 6 | *Latilactobacillus sakei,, Latilactobacillus curvatus, Lactiplantibacillus plantarum, Enterococcus,* family *Lactobacillaceae, genus Pediococcu, Staphylococcus* | 733 | *Lacticaseibacillus thailandensis, Paralactobacillus acidilactici, Latilactobacillus sakei*, et al. | (Carmen et al., 2019) |
|  | fermented ham | 4 | *Micrococcaceae*,Gram-positive, catalase-positive cocci, yeast, moulds | 9 | *Staphylococcus, Bacillus, Cellulosimicrobium, Penicillium commune, Aspergillus fumigatus, Sclerotinia sclerotiorum, Eurotium athecium, Moniliella mellis, Penicillium commune* | (Tu et al., 2010; Martinez-Onandi et al., 2017; Martinez-Onandi et al., 2019; Mu et al., 2020) |
|  |  | 3 | *Micrococcaceae*, moulds and yeasts | 19 | *Ascomycota, Cyanobacteria, Actinobacteria, Bacteroidetes, Basidiomycota, Psychrobacter, uncultured bacterium, Acinetobacter, Ochrobactrum, Staphylococcus, Macrococcus, Cobetia, Chromohalobacter, Kushneria, Debaryomyces, Aspergillus, Yamadazyma, Candida, Penicillium* |  |
| **Fermented condiment** | Shanxi vinegar | 9 | *Pantoea,* family *Lactobacillaceae, genus Pediococcus and Lactobacillus, Lactococcus, Rhizobium, Acetobacter, Komagataibacter, Kroppenstedia, Acetobacter* | 17 | *Staphylococcus gallinarum, Pantoea agglomerans, Leuconostoc holzapfelii, L. holzapfelii, Latilactobacillus sakei, L. sakei, Staphylococcus epidermidis, Escherichia, Shigella, Brenneria* | (Zhu et al., 2018a) |
| **Fermented alcoholic beverages** | Traditional Japanese Sake | 4 | *Aspergillus*, LAB, sokujo-moto, yamahai-moto | 29 | *Firmicutes, Proteobacteria, Tenericutes, Bacteroidetes, Actinobacteria, cyanobacteria,* family *Lactobacillaceae, genus lactobacillus, acetobacter, rhizobia, pine bacillus, mycelia, pseudomonas, Streptomyces, lactococcus, xanthomonas, pyrococcus, gundam, helicobacter py*lori, erythrococcus, *Candidatus_Hepatoplasma, Ralstonia,Lacto bacillus, phyllobiella, Pantea, Pediocus, rhizobia, inulinus, Kroppenstedia* | (Atsushi et al., 2018) |
| **Other fermented foods** | Traditional Irish ciderfermentations | 1 | yeast | 20 | *Saccharomyces cerevisiae NCYC 76, Saccharomyces cerevisiae BM1, Hanseniaspora uvarum BM34, Hanseniaspora uvarum NCYC 2739, Brettanomyces bruxellensis BM 23, Brettanomyces bruxellensis NCYC 2818, Brettanomyces anomalus BM11, Brettanomyces anomalus NCYC 749, Metschinikowia pulcherrima BM 33, Metschinikowia pulcherrima NCYC 373, Debaromyces polymorphus BM53, Debaromyces polymorphus NCYC 947, Pichia fermentas BM43, Pichia fermentas NCYC 562, Pichia guilliermondii BM5, Pichia guilliermondii NCYC 443, Pichia anomola BM 19, Pichia anomola NCYC, Saccharomycodes ludwigii BM 26, Saccharomycodes ludwigii NCYC 734* |  |

^* The names of the genus^ *^Lactobacillus^* ^were designated based on the taxonomy proposed by Zheng et al.(Zheng et al., 2020).^

**Supplementary Tables 2** The succession of microflora in different fermentation stages in fermented foods^*^

| **Fermented foods** | **Subsidiary fermented foods** | **Fermentation prophase** | | **Fermentation metaphase** | | **Fermentation anaphase** | | **References** |
| --- | --- | --- | --- | --- | --- | --- | --- | --- |
|  |  | **Quantities** | **Microbial communities (including beneficial and harmful strains)** | **Quantities** | **Microbial communities (including beneficial and harmful strains)** | **Quantities** | **Microbial communities (including beneficial and harmful strains)** |  |
| **Fermented dairy products** | Yogurt and sour cream | 5 | **Beneficial strains:** *Lacticaseibacillus paracasei. Paracasei, Streptococcus thermophiles,* *Lactobacillus delbrueckii* subsp*. bulgaricus, Lactobacillus delbrueckii sp* | 16 | **Beneficial strains:** *Lactobacillaceae, lactobacilli, Lactobacillus delbrueckii* subsp*. bulgaricus, Bifidobacteria, Lactobacillus paracasei sub sp., Bifidobacterium animalis, Lactobacillus delbrueckii, Streptococcus thermophilus, Lacticaseibacillus casei, Strep. thermophilus*  **Harmful strains:** *Escherichia coli, Staphylococcus aureus, Listeria monocytogenes, Salmonella typhimurium, Shigella flexneri* | 30 | **Beneficial strains:** *Lactobacillus, Lactobacillus delbrueckii* subsp*. bulgaricus, Lacticaseibacillus paracasei* subsp*., Streptococcus thermophilus, S. salivariussub sp. thermophilus, Streptococcus acidominimus, bifidobacteria, Lacticaseibacillus casei, Lactobacillus delbrueckii* subsp*. lactis, Bifidobacterium animalis, Lactobacillus delbrueckii, Firmicutes, Actinobacteria, Verrucomicrobia, Saccharomyces, Vanderwaltozyma, Dekkera, Pichia, Clavispora, Galactomyces, Lacticaseibacillus rhamnosus, Escherichia coli, Salmonella typhimurium, Strep. thermophilus*  **Harmful strains:** *Proteobacteria, Bacteroidetes, Staphylococcus aureus, Listeria monocytogenes, Shigella flexneri* | (Angelakis et al., 2011; Haiyan et al., 2015; Li et al., 2017; Yamauchi et al., 2018) |
|  | Cheese | 2 | **Beneficial strains:** Yeast, LAB | 24 | **Beneficial strains:** *Lactobacillus delbrueckii* subsp*. lactis, Yeast, Agrococcus casei, Lacticaseibacillus casei, Latilactobacillus curvatus, Marinilactibacillus, Debaryomyces, Pichia, Torulaspora, cetic acid bacteria, Lactobacillus delbrueckii* subsp*. lactis, Lentilactobacillus kefiri*  **Harmful strains:** *Brevibacterium, Acinetobacter, Corynebacteri, umStaphylococcus equorum, Bavariicoccusseileri, Alcaligenes faecalis, Corynebacterium variabile, Staphylococcus, Clostridiisalibacte, Pseudoclavibacter, Alkalibacterium, Trichosporon, Candida* | 42 | **Beneficial strains:** *yeast, Lactobacillus delbrueckii* subsp*. lactis, Agrococcus casei, Debaryomyces, Marinilactibacillus, Lactobacillales, Pichia, Torulaspora, Marinilactibacillus psychrotolerans, Corynebacterium casei, Debaryomyceshansenii, lactissubsp.lactis, Bacillus licheniformis, Bacillus pumilus, Bacillus subtilis, Enterococcus faecalis, Enterococcus faecium, cetic acidbacteria, Lactobacillus delbrueckii* subsp*. lactis, Lentilactobacillus kefiri*  **Harmful strains:** *Alcaligenes faecalis, Fusobacterium, Candida, Bavariicoccusseileri, Psychrobacter, Acinetobacter,umStaphylococcus equorum, Brevibacterium, Pseudomonas sp, Chryseobacterium, Citrobacter freundii, Enterobacter cloacae, Serratia fonticola, Klebsiella oxytoca,Alkalibacterium, Clostridiisalibacte, Trichosporon, Enterococcus italicus, Staphylococcus,Pseudoclavibacter, Staphylococcus equorum* | (Mei et al., 2014; Ryssel et al., 2015; Vendula et al., 2018; Bertani et al., 2020) |
| **Fermented vegetable** | Kimchi | 9 | **Beneficial strains:**  family *Lactobacillaceae, genus Lactobacillales, Weisella* and *Leuconostoc, Lactococcus, Rhizobium, Bacillus and Pantoea*  **Harmful strains:** *Pseudomonas, Flavobacterium* | 1 | **Beneficial strains:** family *Lactobacillaceae, genus Leuconostoc* | 3 | **Beneficial strains:** *Lactobacillus, Leu. gasicomitatum and Leu. gelidum* | (Park et al., 2012; Jeong et al., 2013a; Jeong et al., 2013b) |
|  | Chinese sauerkraut | 11 | **Beneficial strains:** *Lactobacillus acidophilus, Limosilactobacillus fermentum, Lactobacillus gasseri,* *Lactobacillus plantarum, Lactobacillus jensenii, E. faecalis, L. lactis, Leuconostoc and Weissella*  **Harmful strains:** *Proteobacteria, Bacteroidetes* | 4 | **Beneficial strains:** *Limosilactobacillus fermentum, Latilactobacillus sakei, Lactobacillus plantarum, Lysinibacillus*  sp., | 5 | **Beneficial strains:** *Lactobacillus plantarum, Lacticaseibacillus casei, Loigolactobacillus coryniformis, Pediococcus*  **Harmful strains:** *Pseudomonas* sp. | (Xiong et al., 2012; Wu et al., 2015; Liang et al., 2018a; Liang et al., 2018b) |
| **Fermented meat products** | Fermented ham and sausage | 10 | **Beneficial strains:** *Macrococcus, Kushneria, Chromohalobacter, Cobetia, Staphylococcus,* family *Lactobacillaceae, genus Leuconostoc spp., Weissella spp. and Lactococcus spp*  **Harmful strains:** *Psychrobacter, Enterococcus spp.* | 7 | **Beneficial strains:** *Chromohalobacter, Cobetia, Staphylococcus*  **Harmful strains:** *Acinetobacter, Brochothrix, Pseudomonas, Psychrobacter* | 7 | **Beneficial strains:** *Kushneria, Chromohalobacter, Cobetia, Staphylococcus, Lactobacillus*  **Harmful strains:** *Psychrobacter, Acinetobacter* | (Mrkonjic Fuka et al., 2020; Mu et al., 2020) |
|  | Vacuum-packaged smoked bacon | 6 | **Beneficial strains:** *Macrococcus, Kocuria,*  family *Lactobacillaceae, genus Lactobacillus, Leuconostoc*  **Harmful strains:** *Brochothrix, Vibrio* | 5 | **Beneficial strains:** family *Lactobacillaceae, genus Leuconostoc,* and *Lactobacillus, Serratia*  **Harmful strains:** *Vibrio, Carnobacterium* | 7 | **Beneficial strains:** family *Lactobacillaceae, genus Lactobacillus, Leuconostoc, Rahnella, Lactococcus, Serratia*  **Harmful strains:** *Carnobacterium, Vibrio* | (Li et al., 2019) |
|  | Salamis | 9 | **Beneficial strains:** *Leuconostoc mesenteroides, Leuconostoc carnosum, Latilactobacillus sakei,*  **Harmful strains:** *Gammaproteobacteria phylum, Moraxellaceae family, Acinetobacter, Pseudomonas, Carnobacterium, Enterococcus* | 10 | **Beneficial strains:** *Gammaprotcobacteria, Photobactericm, Other Mornaxellaceae, Other LAB, Eaterococcus, Carmobacterium Staphoyiococcus*  **Harmful strains:** *Pseudomonas, Acinetobacter* | 9 | **Beneficial strains:** *Gammaprotcobacteria, Photobactericm, Other Mornaxellaceae, Other LAB, Eaterococcus, Carmobacterium Staphoyiococcus*  **Harmful strains:** *Pseudomonas, Acinetobacter* | (Settanni et al., 2020) |
|  | Spanish-type chorizo | 4 | **Beneficial strains:** *Firmutes, Proteobacteria enterobacteriales, Pseudomonadales acillales,* family *Lactobacillaceae, genus Lactobacillales* | 6 | **Beneficial strains:** family *Lactobacillaceae, genus Lactobacillus* (61.9%), *Pseudomonas* (3%), and *Streptococcus* (12%)  **Harmful strains:** *Acinetobacter* (4%), *Bacillus* (3%), *Brochothrix* (1%) | 29 | **Beneficial strains:** Non abundant taxa *Podiococcus, Anoxybacilus, Paenibacilus, Staphlococcus, Brovibacdllus, Vagococcus,* family *Lactobacillaceae, genus Leuconostoc, Lactobaalus and Weissella, Camobacterium, Entorocoocus,  Lactococcus, Brochothnx,  Bacilus, Streptococcus, Klebsiella, Doltia, Ochrobactum, Providencia,  Halomonas, Rahnela, Stenotrophomonas, Serratia, Pantooa,  Shewanela, Enterobactor, Psoudomonas*  **Harmful strains:** *Psychrobacter, Acinetobacter* | (Carmen et al., 2018) |
|  | Normal raw pork sausage | 6 | **Beneficial strains:** *Latilactobacillus sakei* (57%) and *Latilactobacillus curvatus* (14%).  **Harmful strains:** *S. saprophyticus* (61%), *S. equorum* (28%), *S. xylosus* (11%) and *Carnobacterium* sp. (29%) | 5 | **Beneficial strains:** *Latilactobacillus sakei* (77%), *Latilactobacillus curvatus* (7%), *L. delbrueckii subsp. lactis* (8%),  **Harmful strains:** *S. saprophyticus*, *Carnobacterium* sp (8%) | 6 | **Beneficial strains:** *Latilactobacillus sakei* (79%), *Latilactobacillus curvatus* (14%), *Lacticaseibacillus paracasei* (7%)  **Harmful strains:** *S. xylosus* (28%), *S.equorum* (36%), *S.saprophyticus* (36%), | (Charmpi et al., 2020) |
|  | Dark-firm-dry (DFD) pork sausage | 10 | **Beneficial strains:** *S. quorum* (61%), *S. epidermidis* (2%), *S. sciuri* (2%), *S. capitis* (2%), *Latilactobacillus sakei* (18%), *Latilactobacillus curvatus* (39%), *P. agglomerans* (1%)  **Harmful strains:** *S. xylosus* (13%), *S. saprophyticus* (20%), *Carnobacterium* sp. (42%) | 7 | **Beneficial strains:** *M. caseolyticus* (43%), *Latilactobacillus sakei* (22%), *Latilactobacillus curvatus* (69%), *E. faecalis* (9%)  **Harmful strains:** *S. xylosus* (29%), *S. equorum* (21%), *S. saprophyticus* (7%) | 9 | **Beneficial strains:** *M. caseolyticus* (18%), *Latilactobacillus sakei* *i* (44%), *Curvatus* (23%), *L.rhamnosus* (13%), *H. alvei* (10%), *M. morganii* (10%)  **Harmful strains:** *S. xylosus* (28%), *S. equorum* (27%), *S. saprophyticus* (27%) |  |
| **Fermented bean products** | Soybean paste | 5 | **Beneficial strains:** *Penicillium chrysogenum, Aspergillus flavus, A. oryzae, Cladosporium oxysporum, and Pyrenophora phaeocome* | 4 | **Beneficial strains:** *Pichia triangularis, Saccharomyces cerevisae, Malassezia globose, Zygosaccharomyces rouxii* | 9 | **Beneficial strains:** *B. Sonorensis, B. lichemiformis, Weissella cibaria and Tetragenococcus halopilus*  **Harmful strains:** *Bacillus sonorensis, Bacillus licheniformis, Bacillus velezensis, Enterococcus lactis, Carnobacterium maltaromaticum* | (Lee et al., 2017; Chun et al., 2020) |
|  | Fermented mung beans Fermented Mung Beans | 1 | **Beneficial strains:** LAB | 1 | **Beneficial strains:** *Monomonas butyricum* | 0 | NA | (Chao et al., 2013) |
| **Fermented condiment** | Shanxi vinegar | 3 | **Beneficial strains:** family *Lactobacillaceae, genus Lactococcus,*  *Rhizobia*  **Harmful strains:** *Pantoea* | 1 | **Beneficial strains:** LAB | 3 | **Beneficial strains:** *Acetobacter*, *Komagataeibacter*, *Kroppenstedtia* | (Zhu et al., 2018b) |
| **Fermented alcoholic beverages** | Traditional Japanese Sake | 1 | **Beneficial strains:** family *Lactobacillaceae, genus Lactobacillus* | 1 | **Beneficial strains:** *Latilactobacillus sakei* | 1 | **Beneficial strains:** *Latilactobacillus sakei* | (Takashi, 2018) |

^* The names of the genus^ *^Lactobacillus^* ^were designated based on the taxonomy proposed by Zheng et al.(Zheng et al., 2020).^

# References

Akiyama, K., Horita, K., Sakamoto, T., Satozono, H., Takahashi, H., and Goda, Y. (2019). Monitoring the Progress of Lactic Acid Fermentation in Yogurt Manufacturing Using Terahertz Time-Domain–Attenuated Total-Reflection Spectroscopy. *Journal of Infrared, Millimeter, and Terahertz Waves*.

Angelakis, E., Million, M., Henry, M., and Raoult, D. (2011). Rapid and Accurate Bacterial Identification in Probiotics and Yoghurts by MALDI-TOF Mass Spectrometry. *J Food Sci* 76(8)**,** p.198-202.

Anna, Reale, Rocco, G., Ianniello, Felicia, et al. (2016). Effect of respirative and catalase-positive Lactobacillus casei adjuncts on the production and quality of Cheddar-type cheese. *International Dairy Journal*.

Atsushi, T., Miyuki, K., Koji, T., Toshiki, E., and Takashi, K. (2018). Robust Domination of Lactobacillus sakei in Microbiota During Traditional Japanese Sake Starter Yamahai-Moto Fermentation and the Accompanying Changes in Metabolites. *Current Microbiology*.

Bertani, G., Levante, A., Lazzi, C., Bottari, B., Gatti, M., and Neviani, E. (2020). Dynamics of a natural bacterial community under technological and environmental pressures: The case of natural whey starter for Parmigiano Reggiano cheese. *Food Research International* 129.

Carmen, Juárez-Castelán, Israel, García-Cano, Alejandra, Escobar-Zepeda, et al. (2018). Evaluation of the bacterial diversity of Spanish-type chorizo during the ripening process using high-throughput sequencing and physicochemical characterization.

Carmen, Juárez-Castelán, Israel, García-Cano, Alejandra, Escobar-Zepeda, et al. (2019). Evaluation of the bacterial diversity of Spanish-type chorizo during the ripening process using high-throughput sequencing and physicochemical characterization. *Meat science*.

Chao, S.H., Huang, H.Y., Chang, C.H., Yang, C.H., Cheng, W.S., Kang, Y.H., et al. (2013). Microbial Diversity Analysis of Fermented Mung Beans (Lu-Doh-Huang) by Using Pyrosequencing and Culture Methods. *Plos One* 8(5)**,** e63816-.

Charmpi, C., Van der Veken, D., Van Reckem, E., De Vuyst, L., and Leroy, F. (2020). Raw meat quality and salt levels affect the bacterial species diversity and community dynamics during the fermentation of pork mince. *Food Microbiol* 89**,** 103434. doi: 10.1016/j.fm.2020.103434.

Chettri, R., and Tamang, J.P. (2015). Bacillus species isolated from tungrymbai and bekang, naturally fermented soybean foods of India. *Int J Food Microbiol* 197**,** 72-76. doi: 10.1016/j.ijfoodmicro.2014.12.021.

Chun, B.H., Kim, K.H., Jeong, S.E., and Jeon, C.O. (2020). The effect of salt concentrations on the fermentation of doenjang, a traditional Korean fermented soybean paste. *Food Microbiol* 86**,** 103329. doi: 10.1016/j.fm.2019.103329.

da Costa, M.P., Frasao Bda, S., Lima, B.R., Rodrigues, B.L., and Conte Junior, C.A. (2016). Simultaneous analysis of carbohydrates and organic acids by HPLC-DAD-RI for monitoring goat's milk yogurts fermentation. *Talanta* 152**,** 162-170. doi: 10.1016/j.talanta.2016.01.061.

Geeraerts, W., De Vuyst, L., and Leroy, F. (2019). Mapping the dominant microbial species diversity at expiration date of raw meat and processed meats from equine origin, an underexplored meat ecosystem, in the Belgian retail. *Int J Food Microbiol* 289**,** 189-199. doi: 10.1016/j.ijfoodmicro.2018.09.019.

Haiyan, Xu, Wenjun, Liu, Qimu, Gesudu, et al. (2015). Assessment of the bacterial and fungal diversity in home-made yoghurts of Xinjiang, China by pyrosequencing. *Journal of the Science of Food & Agriculture*.

Han, X., Zhang, L., Yu, P., Yi, H., and Zhang, Y. (2014). Potential of LAB starter culture isolated from Chinese traditional fermented foods for yoghurt production. *International Dairy Journal* 34(2)**,** 247-251. doi: 10.1016/j.idairyj.2013.09.007.

Jeong, S.H., Jung, J.Y., Lee, S.H., Jin, H.M., and Jeon, C.O. (2013a). Microbial succession and metabolite changes during fermentation of dongchimi, traditional Korean watery kimchi. *Int J Food Microbiol* 164(1)**,** 46-53. doi: 10.1016/j.ijfoodmicro.2013.03.016.

Jeong, S.H., Lee, H.J., Jung, J.Y., Lee, S.H., Seo, H.-Y., Park, W.-S., et al. (2013b). Effects of red pepper powder on microbial communities and metabolites during kimchi fermentation. *Int J Food Microbiol* 160(3)**,** 252-259. doi: 10.1016/j.ijfoodmicro.2012.10.015.

Jung, J.Y., Lee, S.H., Jin, H.M., Hahn, Y., Madsen, E.L., and Jeon, C.O. (2013). Metatranscriptomic analysis of lactic acid bacterial gene expression during kimchi fermentation. *Int J Food Microbiol* 163(2-3)**,** 171-179. doi: 10.1016/j.ijfoodmicro.2013.02.022.

Lee, S., Lee, S., Singh, D., Oh, J.Y., Jeon, E.J., Ryu, H.S., et al. (2017). Comparative evaluation of microbial diversity and metabolite profiles in doenjang, a fermented soybean paste, during the two different industrial manufacturing processes. *Food Chem* 221**,** 1578-1586. doi: 10.1016/j.foodchem.2016.10.135.

Li, C., Song, J., Kwok, L.Y., Wang, J., Dong, Y., Yu, H., et al. (2017). Influence of Lactobacillus plantarum on yogurt fermentation properties and subsequent changes during postfermentation storage. *J Dairy Sci* 100(4)**,** 2512-2525. doi: 10.3168/jds.2016-11864.

Li, X., Li, C., Ye, H., Wang, Z., Wu, X., Han, Y., et al. (2019). Changes in the microbial communities in vacuum-packaged smoked bacon during storage. *Food Microbiol* 77**,** 26-37. doi: 10.1016/j.fm.2018.08.007.

Liang, H., Chen, H., Zhang, W., Yu, C., Ji, C., and Lin, X. (2018a). Investigation on microbial diversity of industrial Zhacai paocai during fermentation using high-throughput sequencing and their functional characterization. *LWT***,** S0023643818301269.

Liang, H., Yin, L., Zhang, Y., Chang, C., and Zhang, W. (2018b). Dynamics and diversity of a microbial community during the fermentation of industrialized Qingcai paocai, a traditional Chinese fermented vegetable food, as assessed by Illumina MiSeq sequencing, DGGE and qPCR assay. *Annals of Microbiology*.

Liang, R., Huang, J., Wu, X., Xu, Y., Fan, J., Wu, C., et al. (2019). Characterizing the metabolites and the microbial communities of the soy sauce mash affected by temperature and hydrostatic pressure. *Food Res Int* 123**,** 801-808. doi: 10.1016/j.foodres.2019.06.002.

Liu, D., and Tong, C. (2017). Bacterial community diversity of traditional fermented vegetables in China. *LWT* 86**,** 40-48. doi: 10.1016/j.lwt.2017.07.040.

Martinez-Onandi, N., Castioni, A., San Martin, E., Rivas-Canedo, A., Nunez, M., Torriani, S., et al. (2017). Microbiota of high-pressure-processed Serrano ham investigated by culture-dependent and culture-independent methods. *Int J Food Microbiol* 241**,** 298-307. doi: 10.1016/j.ijfoodmicro.2016.11.001.

Martinez-Onandi, N., Sanchez, C., Nunez, M., and Picon, A. (2019). Microbiota of Iberian dry-cured ham as influenced by chemical composition, high pressure processing and prolonged refrigerated storage. *Food Microbiol* 80**,** 62-69. doi: 10.1016/j.fm.2019.01.002.

Mei, J., Guo, Q., Yan, W., Li, Y., and Ali, A.A. (2014). Microbial Diversity of a Camembert-Type Cheese Using Freeze-Dried Tibetan Kefir Coculture as Starter Culture by Culture-Dependent and Culture-Independent Methods. *Plos One* 9(10)**,** e111648-.

Mrkonjic Fuka, M., Tanuwidjaja, I., Zgomba Maksimovic, A., Zunabovic-Pichler, M., Kublik, S., Hulak, N., et al. (2020). Bacterial diversity of naturally fermented game meat sausages: Sources of new starter cultures. *LWT* 118**,** 108782. doi: 10.1016/j.lwt.2019.108782.

Mu, Y., Su, W., Mu, Y., and Jiang, L. (2020). Combined Application of High-Throughput Sequencing and Metabolomics Reveals Metabolically Active Microorganisms During Panxian Ham Processing. *Front Microbiol* 10. doi: 10.3389/fmicb.2019.03012.

Park, E.J., Chun, J., Cha, C.J., Park, W.S., Jeon, C.O., and Bae, J.W. (2012). Bacterial community analysis during fermentation of ten representative kinds of kimchi with barcoded pyrosequencing. *Food Microbiol* 30(1)**,** 197-204. doi: 10.1016/j.fm.2011.10.011.

Pini, F., Aquilani, C., Giovannetti, L., Viti, C., and Pugliese, C. (2020). Characterization of the microbial community composition in Italian Cinta Senese sausages dry-fermented with natural extracts as alternatives to sodium nitrite. *Food Microbiol* 89**,** 103417. doi: 10.1016/j.fm.2020.103417.

Ryssel, M., Johansen, P., Al-soud, W.A., S?rensen, S., Arneborg, N., and Jespersen, L. (2015). Microbial diversity and dynamics throughout manufacturing and ripening of surface ripened semi-hard Danish Danbo cheeses investigated by culture-independent techniques. *Int J Food Microbiol*.

Schirmer, B.C., Heir, E., Moretro, T., Skaar, I., and Langsrud, S. (2013). Microbial background flora in small-scale cheese production facilities does not inhibit growth and surface attachment of Listeria monocytogenes. *J Dairy Sci* 96(10)**,** 6161-6171. doi: 10.3168/jds.2012-6395.

Settanni, L., Barbaccia, P., Bonanno, A., Ponte, M., Di Gerlando, R., Franciosi, E., et al. (2020). Evolution of indigenous starter microorganisms and physicochemical parameters in spontaneously fermented beef, horse, wild boar and pork salamis produced under controlled conditions. *Food Microbiol* 87**,** 103385. doi: 10.1016/j.fm.2019.103385.

Silva, V.L.M., Costa, M.P., Vieira, C.P., and Conte-Junior, C.A. (2019). Short communication: Biogenic amine formation during fermentation in functional sheep milk yogurts. *J Dairy Sci* 102(10)**,** 8704-8709. doi: 10.3168/jds.2019-16379.

Song, Y.R., Jeong, D.Y., and Baik, S.H. (2015). Effects of indigenous yeasts on physicochemical and microbial properties of Korean soy sauce prepared by low-salt fermentation. *Food Microbiol* 51**,** 171-178. doi: 10.1016/j.fm.2015.06.001.

Sulaiman, J., Gan, H.M., Yin, W.F., and Chan, K.G. (2014). Microbial succession and the functional potential during the fermentation of Chinese soy sauce brine. *Front Microbiol* 5**,** 556. doi: 10.3389/fmicb.2014.00556.

Sun, X., Lyu, G., Luan, Y., Zhao, Z., Yang, H., and Su, D. (2018). Analyses of microbial community of naturally homemade soybean pastes in Liaoning Province of China by Illumina Miseq Sequencing. *Food Research International* 111**,** 50-57. doi: 10.1016/j.foodres.2018.05.006.

Takashi, K. (2018). Robust Domination of Lactobacillus sakei in Microbiota During Traditional Japanese Sake Starter Yamahai-Moto Fermentation and the Accompanying Changes in Metabolites. *Current Microbiology*.

Tu, R.-J., Wu, H.-Y., Lock, Y.-S., and Chen, M.-J. (2010). Evaluation of microbial dynamics during the ripening of a traditional Taiwanese naturally fermented ham. *Food Microbiol* 27(4)**,** 460-467. doi: 10.1016/j.fm.2009.12.011.

Vendula, P., Leona, B., Sabina, P., Irena, N.k., ?árka, H., Khatantuul, P., et al. (2018). Contaminating microorganisms in quark-type cheese and their capability of biogenic amine production. *International Journal of Dairy Technology*.

Wang, X., Zhang, Y., Ren, H., and Zhan, Y. (2018). Comparison of bacterial diversity profiles and microbial safety assessment of salami, Chinese dry-cured sausage and Chinese smoked-cured sausage by high-throughput sequencing. *LWT* 90**,** 108-115. doi: 10.1016/j.lwt.2017.12.011.

Wu, R., Yu, M., Liu, X., Meng, L., Wang, Q., Xue, Y., et al. (2015). Changes in flavour and microbial diversity during natural fermentation of suan-cai, a traditional food made in Northeast China. *Int J Food Microbiol* 211**,** 23-31. doi: 10.1016/j.ijfoodmicro.2015.06.028.

Xiong, T., Guan, Q., Song, S., Hao, M., and Xie, M. (2012). Dynamic changes of lactic acid bacteria flora during Chinese sauerkraut fermentation. *Food Control* 26(1)**,** 178-181.

Yamauchi, R., Maguin, E., Horiuchi, H., Hosokawa, M., and Sasaki, Y. (2018). The critical role of urease in yogurt fermentation with various combinations of Streptococcus thermophilus and Lactobacillus delbrueckii ssp. bulgaricus. *Journal of Dairy Science*.

Yang, C., Zhao, F., Hou, Q., Wang, J., Li, M., and Sun, Z. (2020). PacBio sequencing reveals bacterial community diversity in cheeses collected from different regions. *J Dairy Sci* 103(2)**,** 1238-1249. doi: 10.3168/jds.2019-17496.

Yee, A.L., Maillard, M.-B., Roland, N., Chuat, V., Leclerc, A., Pogačić, T., et al. (2014). Great interspecies and intraspecies diversity of dairy propionibacteria in the production of cheese aroma compounds. *Int J Food Microbiol* 191**,** 60-68. doi: 10.1016/j.ijfoodmicro.2014.09.001.

Yu, J., Mo, L., Pan, L., Yao, C., Ren, D., An, X., et al. (2018). Bacterial Microbiota and Metabolic Character of Traditional Sour Cream and Butter in Buryatia, Russia. *Front Microbiol* 9**,** 2496. doi: 10.3389/fmicb.2018.02496.

Zheng, J., Wittouck, S., Salvetti, E., Franz, C., Harris, H.M.B., Mattarelli, P., et al. (2020). A taxonomic note on the genus Lactobacillus: Description of 23 novel genera, emended description of the genus Lactobacillus Beijerinck 1901, and union of Lactobacillaceae and Leuconostocaceae. *Int J Syst Evol Microbiol* 70(4)**,** 2782-2858. doi: 10.1099/ijsem.0.004107.

Zhu, Y., Zhang, F., Zhang, C., Yang, L., Fan, G., Xu, Y., et al. (2018a). Dynamic microbial succession of Shanxi aged vinegar and its correlation with flavor metabolites during different stages of acetic acid fermentation. *Scientific Reports* 8(1). doi: 10.1038/s41598-018-26787-6.

Zhu, Y., Zhang, F., Zhang, C., Yang, L., Fan, G., Xu, Y., et al. (2018b). Dynamic microbial succession of Shanxi aged vinegar and its correlation with flavor metabolites during different stages of acetic acid fermentation. *Sci Rep* 8(1)**,** 8612. doi: 10.1038/s41598-018-26787-6.

Ziarno, M., and Zareba, D. (2020). The effect of the addition of microbial transglutaminase before the fermentation process on the quality characteristics of three types of yogurt. *Food Sci Biotechnol* 29(1)**,** 109-119. doi: 10.1007/s10068-019-00640-6.
